# An In-vitro Assessment of the Physical and Chemical Properties of Toothbrush Bristle Following Decontamination by Three Different Methods: A Pilot Study

**DOI:** 10.7759/cureus.4992

**Published:** 2019-06-25

**Authors:** Ram Sabarish, Sree Ramya Chaparala, Padma Priya Yelisetty, Balaji SK, Vamsi Lavu, Meenakshi Mohan

**Affiliations:** 1 Periodontology, Faculty of Dental Sciences, Sri Ramachandra Medical College and Research Institute, Chennai, IND; 2 Periodontology, Private Practice, Chennai, IND

**Keywords:** toothbrush, toothbrush decontamination, aerosols, scanning electron microscopy, fourier transform infrared spectroscopy

## Abstract

Background

Toothbrushes are the most commonly used mechanical plaque control methods, and they are said to harbor microorganisms if not stored properly following usage.

Objective

An assessment of the influence of a decontaminating agent (sterile water (control)/chlorhexidine mouthwash/herbal mouthwash) on the properties of toothbrush bristles following storage for 24 hrs by means of scanning electron microscopy (SEM) and Fourier transform infrared spectroscopy (FTIR).

Methodology

The study involves a total of 24 slim soft toothbrushes (six samples per group) with different decontaminating agents: Group 1: Vented container, Group 2: Sterile water, Group 3: 0.2% Chlorhexidine mouthwash, and Group 4: Herbal mouthwash. The toothbrushes were stored in their respective containers for 24 hours, following which, snippets of toothbrush bristles were collected and tested for the evaluation of wear using SEM and the biochemical alteration occurring in the bristles was assessed using FTIR.

Results and conclusion

The Group 2 and Group 3 samples showed significant wear (Score 2) under SEM evaluation, and FTIR spectroscopy evaluation revealed that changes occur in the fingerprint region of infrared spectroscopy owing to chemical bond alteration following decontamination. Considering the benefits we acquire in terms of microbial load reduction, decontamination is recommended, though it might necessitate frequent changing of toothbrushes.

## Introduction

Dental biofilm or dental plaque, a causative factor responsible for the development of dental caries and the initiation of gingival and periodontal diseases, has been an area of interest among researchers for decades. Soon after brushing, a thin layer of salivary proteins (pellicle) accumulates over the tooth surface. The pellicle serves as an adhesive that facilitates the attachment and colonization of numerous micro-organisms. This tenacious microbial layer is referred to as dental plaque. When not cleaned regularly, dental plaque attracts and facilitates the co-aggregation of primary and secondary bacterial colonizers, thus forming a more complex structure that can compete with the native flora of the oral cavity, causing disease.

Effective plaque removal is important for the maintenance of proper oral hygiene. Two types of plaque control methods are: mechanical (tooth brushing, flossing, and scaling) and chemical (mouthwashes). The advent of various oral hygiene aids has contributed significantly to the prevention of oral diseases, with toothbrushes (TB) being the most economical one. When used correctly, TB help maintain optimal levels of oral hygiene. Other auxiliary aids available in the market include interdental brushes, dental floss, and mouthwashes. An assortment of TB such as slim soft TB, charcoal-coated, neem-coated, and biodegradable ones are also available [1].

Various sources of contamination of toothbrushes include the oral cavity itself, the outer environment, contaminated hands, aerosol contamination, and storage containers [2]. The area of the toothbrush in which the tufts are anchored serves as a niche for microbes to colonize [3]. Various strains of bacteria such as Streptococcus mutans, Staphylococcus aureus, Pseudomonas, Lactobacillus, Klebsiella, and Candida species were isolated from TB stored in bathrooms without an attached toilet. Escherichia coli were found in addition to the other micro-organisms when kept in a bathroom with an attached toilet [3-4]. There is a complete lack of awareness in the public regarding toothbrush maintenance so it is of utmost importance to educate them about proper storage, replacement, and disinfection methods. Toothbrushes play a pivotal role in fighting tooth decay but can also potentially lead to dental as well as other systemic diseases, including septicemia, gastrointestinal, cardiovascular, respiratory, and renal problems, if not properly stored and maintained [4]. In 1996, the American Dental Association (ADA) has recommended a change of TB once every three months.

Rinsing with an antibacterial mouthwash before brushing may prevent or decrease how rapidly bacteria build up on toothbrushes. Soaking toothbrushes in an antibacterial mouth rinse (chlorhexidine [5], HiOra (The Himalaya Drug Company, Bangalore, India), or 3% neem) after use has also been studied and a significant decrease in bacterial contamination was observed [6-7]. Recent advancements, such as an ultraviolet (UV) light toothbrush holder (Violight, VIOlight Inc., NY, US), reduce the total colony-forming units by an average of 86% [8], soaking brushes in an ozone-saturated phosphate-buffered saline (PBS) solution was shown to be an effective alternative after a 30-minute exposure [9]. Mouthwash contains active ingredients that act as a decontaminant for toothbrushes, thereby serving as an economical and accessible solution for toothbrush decontamination. Although microbial reduction following the decontamination of the toothbrush has been evaluated, the effect of these decontaminating agents on the toothbrush bristles' physicochemical properties is unknown. Hence, the objective of this study was to evaluate the influence of three decontaminating agents (sterile water, 0.2% chlorhexidine, and commercially available herbal mouthwash) on toothbrush bristle properties by scanning electron microscopy (SEM) and Fourier transform infrared spectroscopy (FTIR).

## Materials and methods

An in-vitro study was conducted in the department of periodontology, Sri Ramachandra University, Chennai. The study design was approved by the institutional review board, and ethical clearance (CSP/17/DEC/62/359) was obtained to proceed with the study. A sample size of 24 toothbrushes was chosen for the present study.

A slim, soft toothbrush (17 times slimmer than the conventional toothbrush) was chosen for the present study, to assess even minute physical and chemical changes of the bristles following the toothbrush decontamination procedure using commercially available herbal mouthwash (HiOra). HiOra is a herbal-based mouthwash consisting of 10 mg Bibhitaki (Terminalia bellirica), 10 mg Nagavalli (Piper betle), 5 mg Pilu (Salvadora persica), 1.6 mg Peppermint satva (Mentha piperita), 0.4 mg Yavani satva (Trachyspermum ammi), 1.2 mg Gandhapura taila (Gaultheria fragrantissima), and 0.2 mg Ela (Elettaria cardomomum).

The study comprised 24 brand new, slim, soft toothbrushes, which were distributed into four groups (one Control Group and three Experimental Groups) of six brushes each. The toothbrushes were kept in the respective containers as below:

Group 1 - Brushes were kept in standardized containers with air vents.

Group 2 - Brushes were kept immersed in containers with sterile water.

Group 3 - Brushes were kept immersed in containers with 0.2% chlorhexidine.

Group 4 - Brushes were kept immersed in containers with commercially available herbal mouthwash.

A total number of 24 containers of equal shape and size were selected. The containers were filled with 60 ml of the respective undiluted solutions, i.e., six containers were filled with 60 ml of sterile water, six containers were filled with 60 ml of 0.2% chlorhexidine, six containers were filled with 60 ml of commercially available herbal mouthwash, and six containers were left empty for the control group. One toothbrush per container was placed in such a way that the heads were completely immersed inside the respective solution. The containers were then placed inside a laminar flow chamber for 24 hours. Following this, the toothbrush heads were analyzed for physical and chemical wear-offs using SEM with energy dispersion spectroscopy (EDS) and FTIR. Scoring of the samples, when viewed under SEM, was done according to the evaluation criteria (Table 1). The alteration in the properties of the toothbrush was compared against the control (Group 1).

**Table 1:** Modified evaluation criteria for scoring SEM Images by Nuss et al. [10]

EVALUATION	SEM EVALUATION CRITERIA
Score 1	New appearance, smooth bristle surface, bristles standing straight and even, no split ends.
Score 2	Slight signs of wear, bristles standing straight and even, irregular bristle ends, little surface roughness, no split ends.
Score 3	Clear signs of wear, lot of surface roughness, fraying and bending, order of tufts not recognizable, slight end splitting.
Score 4	A clear sign of wear, lot of surface roughness, severe fraying and bending, order of tufts no longer recognizable, slight end splitting.

Assessment under SEM

The toothbrush heads were separated under sterile aseptic conditions and labeled. The samples were mounted on the metal stubs and dried in silica gel vacuum desiccators. Then, they were sputter coated with gold and examined under the SEM. Micrographs of the bristle surfaces were obtained at 95x magnification at 10 KV voltage with a eucentric sample stage.

Assessment under FTIR

Toothbrush bristles were severed using a number 11 Bard-Parker blade (Aspen Surgical, MI, US) and forceps under aseptic conditions for FTIR analysis. The bristles were placed directly on top of the crystal at the center of the sample slot, which was 1 cm in diameter. The compression tip was then tightened over the bristles and the crystal, creating a vacuum. Following this, the bristle sample was exposed to different wavelengths of infrared light, ranging from 10,000 to 100 cm^-1^. Some of the radiations were absorbed while some passed through. The wavelengths of the absorbed radiations were measured and displayed electronically as a graphical representation, with the wave numbers on the x-axis and the transmittance on the y-axis. The resulting graph was studied by comparing the various peaks seen in the graph to their respective wave numbers, ranging from 4000 cm^-1^ to 400 cm^-1^. These further relate to the functional groups that are predominantly present in the given sample, thus helping us analyze the chemical composition of the sample.

## Results

SEM

**Figure 1:**
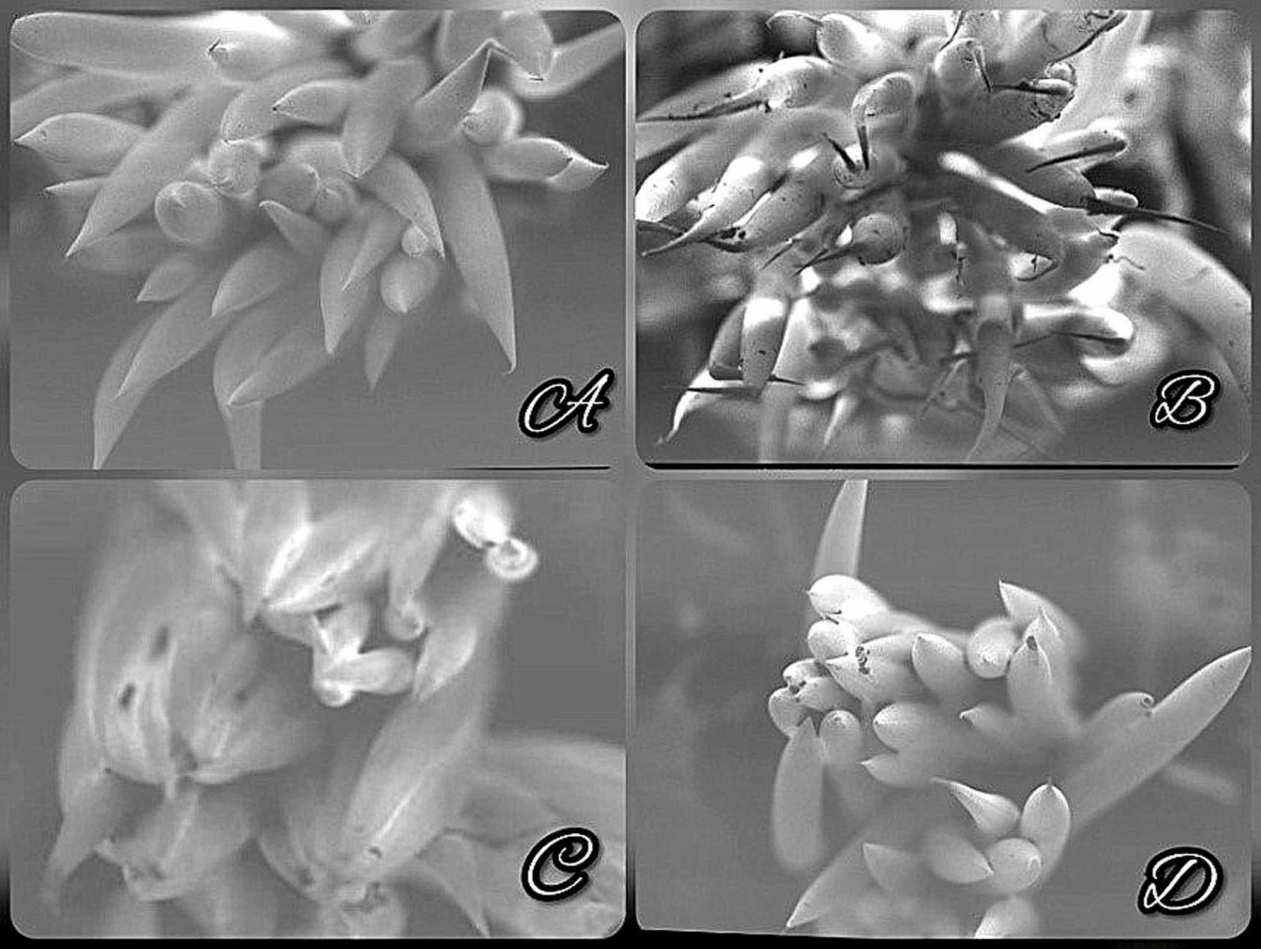
SEM micrographs of toothbrush bristles following 24-hour decontamination. Images A, B, C, and D are micrographs of bristles placed in an air-vented container, distilled water, chlorhexidine, and HiOra mouthwash respectively. SEM: scanning electron microscopy HiOra: The Himalaya Drug Company, Bangalore, India

Scanning electron microscopic assessment of the toothbrush bristles in Group 2 and Group 3 revealed slight wear of the bristles with irregular bristle end rounding, as identified in Figure 1. This corresponds to Score 2 of the Nuss et al. [10] classification. Table 2 depicts the modified SEM scores of individual toothbrush heads in each group. As per data, considerable wear of bristles was found when the toothbrushes are immersed in sterile distilled water and 0.2% chlorhexidine mouthwash.

**Table 2:** Depicts modified SEM scores of each sample SEM: scanning electron microscopy

SAMPLE	GROUP 1	GROUP 2	GROUP 3	GROUP 4
A	1	2	2	1
B	1	3	2	1
C	1	3	2	1
D	1	2	2	1
E	1	2	2	1
F	1	2	2	1

Statistical analysis

An analysis of variance (ANOVA) analysis was done to check for the significant level of difference between the groups using SPSS 20.0 software (SPSS Inc., Chicago, Ill., US). Table 3 depicts a summary of the ANOVA analysis done. Results show that there is a significant (p<0.05) mean difference between the groups.

**Table 3:** Summary of ANOVA Analysis ANOVA: analysis of variance

Intervention	n	Mean±SD	SE	F	P
1.00	6	0	0	42.4981	0.000
2.00	6	2.3333±0.5164	0.2108		
3.00	6	2	0		
4.00	6	0	0		
Source of variation	Sum of Squares	d.f.	Variance		
Between Groups	8.4997	3	2.8332		
Within Groups	1.3333	20	0.0667		

FTIR

**Figure 2:**
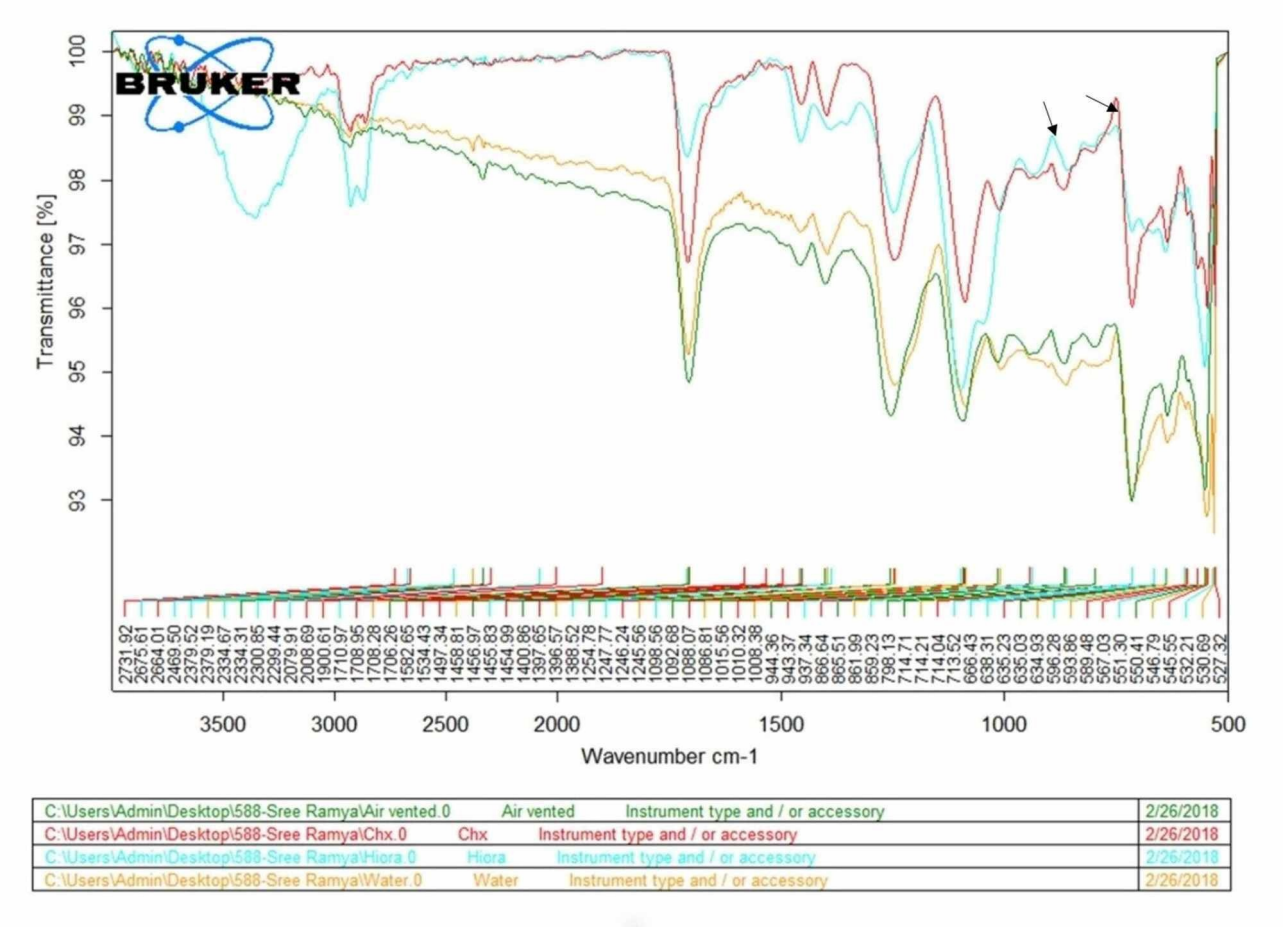
Graphical representation of FTIR analysis FTIR: Fourier transform infrared spectroscopy

An FTIR assessment was done to analyze the chemical changes brought about by the disinfectants (chlorhexidine and herbal mouth wash) on the bristles (Figure 2). No such significant alterations were observed on comparing the different groups. Minimal changes were observed in Group 2 and 3 bristles belonging to the fingerprint region of the IR spectrum (Table 4). These subtle changes are suggestive of bending of the single bond region (C-O, C-C, C-N) of the nylon molecule.

**Table 4:** Depicts the presence or absence of chemical changes in the toothbrush bristles *Y: Presence; N: Absence

SAMPLE	GROUP 1	GROUP 2	GROUP 3	GROUP 4
A	N	Y	Y	N
B	N	Y	Y	N
C	N	Y	Y	N
D	N	Y	Y	N
E	N	Y	Y	N
F	N	Y	Y	N

## Discussion

Toothbrush bristles are a source of bacterial cross-infection due to their storage in areas with high microbial content in the environment. Hence, in the recent past, a considerable amount of research has been done to assess different methods of decontamination.

Chemical agents, such as cetylpyridinium chloride [11], 1.0% sodium hypochlorite, chlorhexidine, Dettol, essential oil mouthwash, and 3% hydrogen peroxide [12], have been shown to be efficacious in reducing the microbial contamination of toothbrushes. However, there is scant literature on the influence of these chemical agents on toothbrush bristle properties. This assumes importance after reports in the literature wherein clinical trials evaluating the plaque removal efficacy of two- to three-month-old tooth-brushes versus new brushes [13-14] have reported three-month-old toothbrushes to be associated with more tooth wear with less plaque removal efficacy.

This pilot study attempts to assess the physico-chemical properties of toothbrush bristles by using SEM with EDS and FTIR techniques following 24-hour decontamination with 0.2% chlorhexidine and commercially available herbal mouthwash HiOra.

SEM with EDS and FTIR were the techniques of choice in the present study. SEM is a method used widely for the morphological assessment of both hard and soft materials. An analysis of the surface morphology can be done at a micrometer to nanometer level for the selected specimen. The use of EDS allows the assessment of the presence and relative abundance of the elements that make up the surface of the specimen under study.

The observation by the SEM examination was that a significant difference was observed in terms of bristle wear (score of Nuss et al. [10] classification) among the distilled water group and the 0.2% chlorhexidine group. Since no previous published literature exists, a comparison of the data is not feasible at present.

FTIR is a method of infrared spectroscopy that can detect molecular vibrations based on which functional groups can be identified. Specific infrared absorptions bands that are characteristic of the functional groups are detected by FTIR, and this method was used in the present study to identify changes in the chemical properties of the toothbrush bristles after decontamination with different agents.

In the present study, the SEM findings were also supported by FTIR observations wherein the presence of chemical changes was seen in the distilled water and 0.2% chlorhexidine group.

An unexpected finding was the lack of changes in the physico-chemical properties of bristles when decontamination was done with HiOra®. This could possibly be explained by the HiOra® composition, which is a mixture of herbal oils. The oil mixture may not induce changes in the nylon, as it is proven that nylon resins are extremely resistant to oils.

In contrast, nylon is less resistant to water and it interacts readily with water molecules and absorbs it. This has deleterious effects over time, with a reduction in the tensile strength, modulus of elasticity, and impact strength following its repeated interactions with water. This explains the above-mentioned changes in the distilled water group and the chlorhexidine group and the lack of similar changes in the herbal mouthwash group.

To summarize, the findings of the present study provide evidence that bristle properties change after decontamination with commonly used agents such as 0.2% chlorhexidine mouth wash and distilled water. Another important finding is that the herbal formulation has no deleterious effects on the bristle properties. The limitations of this study include the duration of decontamination (24-hour period). Since 0.2% chlorhexidine has the property of substantivity, continuous immersion may not be needed for the decontamination of bristles. No comparative literature exits to the best of our knowledge, and this may be the first study to have assessed the changes in the properties of toothbrush bristles after decontamination.

Future studies need to be done evaluating the efficacy of HiOra on the decontamination of bristles, as the herbal formulation has shown promising results by not affecting the bristle properties.

## Conclusions

To conclude, the current study suggests that toothbrush decontamination is necessary but when performed regularly, the patient might have to change the brush more frequently in order to prevent excessive mechanical wear of the tooth due to bristle property alterations. On comparing the various methods of toothbrush decontamination, the use of herbal mouthwash was found to have the least influence on toothbrush bristle properties.
